# Pgam5-mediated PHB2 dephosphorylation contributes to endotoxemia-induced myocardial dysfunction by inhibiting mitophagy and the mitochondrial unfolded protein response

**DOI:** 10.7150/ijbs.85767

**Published:** 2023-08-28

**Authors:** Chen Cai, Ziying Li, Zemao Zheng, Zhongzhou Guo, Qian Li, Shuxian Deng, Nengxian Shi, Qing Ou, Hao Zhou, Zhigang Guo, Zhongqing Chen, Hang Zhu

**Affiliations:** 1 Department of Critical Care Medicine, Nanfang Hospital, Southern Medical University, Guangzhou 510515, China.; 2 Department of Critical Care Medicine, The First School of Clinical Medicine, Southern Medical University, Guangzhou 510515, China.; 3 Department of Respiratory and Critical Care Medicine, Nanfang Hospital, Southern Medical University, Guangzhou 510515, China.; 4 Department of Pharmacy, Zhujiang Hospital, Southern Medical University, Guangzhou, 510280, China.; 5 The Second Affiliated Hospital of Guangzhou Medical University, Guangzhou, China.; 6 School of Medicine, University of Rochester Medical Center Rochester, Rochester, NY 14642, United States.; 7 Department of Cardiology, Huiqiao Medical Center, Nanfang Hospital, Southern Medical University, Guangzhou 510515, China.; 8 Senior Department of Cardiology, The Sixth Medical Center of People's Liberation Army General Hospital, Beijing, China.

**Keywords:** Pgam5, PHB2, endotoxemia-related cardiac dysfunction

## Abstract

Numerous mitochondrial abnormalities are reported to result from excessive inflammation during endotoxemia. Prohibitin 2 (PHB2) and phosphoglycerate mutase 5 (Pgam5) have been associated with altered mitochondrial homeostasis in several cardiovascular diseases; however, their role in endotoxemia-related myocardial dysfunction has not been explored. Our experiments were aimed to evaluate the potential contribution of Pgam5 and PHB2 to endotoxemia-induced mitochondrial dysfunction in cardiomyocytes, with a focus on two endogenous protective programs that sustain mitochondrial integrity, namely mitophagy and the mitochondrial unfolded protein response (UPR^mt^). We found that PHB2 transgenic mice are resistant to endotoxemia-mediated myocardial depression and mitochondrial damage. Our assays indicated that PHB2 overexpression activates mitophagy and the UPR^mt^, which maintains mitochondrial metabolism, prevents oxidative stress injury, and enhances cardiomyocyte viability. Molecular analyses further showed that Pgam5 binds to and dephosphorylates PHB2, resulting in cytosolic translocation of mitochondrial PHB2. Silencing of Pgam5 or transfection of a phosphorylated PHB2 mutant in mouse HL-1 cardiomyocytes prevented the loss of mitochondrially-localized PHB2 and activated mitophagy and UPR^mt^ in the presence of LPS. Notably, cardiomyocyte-specific deletion of Pgam5 *in vivo* attenuated LPS-mediated myocardial dysfunction and preserved cardiomyocyte viability. These findings suggest that Pgam5/PHB2 signaling and mitophagy/UPR^mt^ are potential targets for the treatment of endotoxemia-related cardiac dysfunction.

## Introduction

Endotoxemia-related myocardial dysfunction, characterized by compromised heart function as a result of an abnormal inflammatory response, is a serious complication of systemic infection induced by lipopolysaccharide (LPS, endotoxin) of gram-negative bacteria 1-3. The primary feature of such condition is a sudden depression of myocardial contractility. Several pathological factors, such as disrupted coronary microcirculation, cardiac calcium overload, mitochondrial oxidative stress, myocardial swelling, and cardiomyocyte death, have been identified as contributors of defective myocardial output during generalized sepsis 4-8. However, the underlying molecular mechanisms have not been fully uncovered, and therefore fluid resuscitation and administration of vasoactive or inotropic agents remain the first-line treatments for patients with endotoxemic cardiomyopathy.

Mitochondrial abnormalities are commonly induced by the excessive inflammatory response characteristic of endotoxemia 9. Impaired oxidative phosphorylation, increased ROS production, altered mitochondrial calcium handling, and mitochondria-related cell death, collectively leading to myocardial metabolic disturbance and cardiac dysfunction, are classically induced by endotoxin exposure 10-14. Accordingly, pharmacological interventions aimed at reducing mitochondrial dysfunction were found to delay the progression of endotoxemic cardiomyopathy and improve patient prognosis 15-17. Notably, injured mitochondria are capable of self-repair through endogenous protective mechanisms such as mitophagy and the mitochondrial unfolded protein response (UPR^mt^) 18-20. Mitophagy is a degradative process which removes non-functional mitochondria, whereas UPR^mt^ defines a mitochondria-controlled nuclear transcriptional program which upregulates protective genes to maintain mitochondrial proteostasis 21-23. Considering that therapeutic strategies targeting mitochondria have a potentially deep impact in the treatment of sepsis-related organ dysfunction, it is worthy of consideration whether stimulation of endogenous mitochondrial protective mechanisms would benefit heart performance during endotoxemia-induced cardiomyopathy.

Prohibitin 2 (PHB2) is a nucleus-encoded protein that plays an indispensable action on maintaining the integrity of the inner mitochondrial membrane (IMM) 24-26. Noteworthy, various mitochondrial activities, including metabolic cycles, mitophagy, and mitochondrial biogenesis, are controlled by PHB2 27-29. Recent reports have highlighted the cardioprotective actions of PHB2 during myocardial fibrosis 30 and heart failure 31, in potential association with activation of mitophagy 32 and improvement in mitochondrial metabolism 31. However, it remains unknown whether there is a cause-effect relationship between PHB2 and UPR^mt^ activation. Although prohibitin 1 (PHB1) overexpression was found to attenuate sepsis-related cardiomyopathy through restoring the activity of the mitochondrial respiratory chain 33, 34, evidence for the precise role of PHB2 in endotoxemia-related myocardial depression is still lacking.

Recent studies have found that several phosphorylation reactions on PHB2 residues determine its intracellular sub-localization 35, 36. Thus, dephosphorylated PHB2 is primarily detected in the cytoplasm, whereas phosphorylated PHB2 localizes in the mitochondria 36-38. PHB2 dephosphorylation induces its translocation from mitochondria to the cytosol 36, 37, 39, 40, resulting in IMM remodeling and activation of apoptosis. In light of this, it is of interest to assess the phosphorylation status of PHB2 during endotoxemia. After exploring potential phosphatases upstream of PHB2, we focused on phosphoglycerate mutase 5 (Pgam5), which was reported to elicit PHB2 dephosphorylation during diabetic cardiomyopathy 41. Using a mouse model of endotoxemic cardiomyopathy, as well as cultured mouse HL-1 cardiomyocytes, the present works highlights a novel mechanism of mitochondrial dysfunction and impaired heart function mediated by Pgam5-dependent PHB2 dephosphorylation in endotoxemic cardiomyocytes.

## Materials and Methods

### Animal

Ten-weeks-old wild type (WT) mice on a mixed background (C57BL/6J and 129/Sv), PHB2 transgenic (PHB2*^Tg^*) mice, and cardiomyocyte-specific Pgam5 knockout (Pgam5*^Cko^*) mice were established based on our previous research. The mouse endotoxemia model was performed via a single intraperitoneal injection of LPS (10 mg/kg). The mice were sacrificed 24 h after the intervention, after echocardiography evaluation, under sodium pentobarbital anesthesia (60 mg/kg). Control mice received a similar volume of PBS.

### H&E staining

Tissue morphology was analyzed with H&E staining. In briefly, paraffin-embedded mouse heart was cut, deparaffinized by a two-step 5-min incubation in xylene at room temperature, and rehydrated by incubation in decreasing percentages of ethanol. After washing with DPBS 1x, slides were incubated for 5 min in Harris hematoxylin (Merck Millipore), washed with tap water, and incubated another 5 min with eosin (Merck Millipore) at room temperature. Finally, the heart sections were washed, dehydrated, and finally mounted with DPX (Sigma-Aldrich).

### Electron microscopy (EM)

Heart sections were treated with 2.5% glutaraldehyde (pH 7.4) for 1 hour, washed in phosphate buffer (100 mM, CaCl_2_-free), and then treated with 1% osmium tetroxide for 1 hour. Staining was done with 2% uranyl acetate in maleate buffer (pH 5.2) for 1 hour. After rinsing and ethanol dehydration, samples were embedded in Embed812 resin (EMS). Area of interest was captured under a JEM-1400Plus electron microscope (Joel Ltd, Tokyo, Japan) at 80 kV.

### Echocardiography

We used echocardiography with a VisualSonics Vevo 2100 system to evaluate heart function in WT, PHB2*^Tg^*, and Pgam5*^Cko^* mice. First, anesthetization was performed through 3.0% isoflurane. Then, parasternal long-axis images, M-mode images were used to measure myocardial structure and function based on previous studies. Echocardiographic images of the ascending and abdominal aorta were also obtained to quantify their maximum diameters 42. All measurement data are the average of at least three consecutive cardiac cycles.

### RT-qPCR and immunoblotting

RT-qPCR was conducted as previously described. The primers used for RT-qPCR can be found in Supplementary Table 1. Three biological replicates were measured for each condition. Cells and heart tissues were lysed in RIPA containing various components. 50 to 100 g of protein per lane was separated by SDS-PAGE and then transferred to PVDF membranes. PHB2 antibodies (sc-133094, Santa Cruz Biotechnology) was used to probe the levels of PHB2 in HL-1 cells.

### Immunofluorescence and PI staining

Cell samples were fixed in 10% formalin washed in Hank's solution, and blocked using PBS supplemented with 10% goat normal serum. Samples were then washed with PBST and then treated with primary antibodies at 4°C overnight. Subsequently, samples were then incubated with fluorescence-conjugated secondary antibodies. After stained with DAPI, areas of interest were captured by a Zeiss Axio Observer Z1 microscope.

Apoptosis was assessed by propidium iodide (PI) staining using a PI staining Kit (P1304MP, ThermoFisher). Images were acquired using a Zeiss Axio Observer Z1 microscope.

### Analysis of mitochondrial membrane potential

Mitochondrial potential, which represents the state of energy production and cell health, was measured in this study 43. To do so, we used a Mitochondrial Membrane Potential Assay Kit (13296, Cell Signaling Technology, Danvers, MA, USA). This kit utilizes JC-1 staining, a fluorescent dye that specifically accumulates in active and healthy mitochondria. By measuring the fluorescence intensity of JC-1, we were able to assess the mitochondrial potential. To capture the images, we employed a laser scanning confocal microscope, which provides high-resolution and detailed images of the stained mitochondria.

### Gene knockdown and overexpression

Stable Pgam5 knockdown was achieved by a lentiviral-based shRNA system. Target-specific or nontarget control shRNAs (Sigma Aldrich) were subcloned into pLKO.1-Puro vectors. The shRNA plasmids were first transfected into 293FT cells for viral packaging, and the lentivirus-containing supernatant was then collected and used to infect HL-1 cells. Selection of cells expressing Pgam5-targeted shRNA was performed by addition of 0.75 mg/ml of puromycin for 5-7 days. Pgam5 knockdown efficiency was verified at both mRNA and protein levels using, respectively, RT-qPCR and western blotting 44.

To stably overexpress native PHB2 and phosphomimetic PHB2-Ser^39^ (PHB2S39D) and phospho-defective (PHB2S39A) mutant proteins, cDNAs generated from HL-1 cell lines were subcloned into lenti-P2A-blast vectors (generated from lenti-Cas9-blast constructs provided by Dr. Sam Toan, University of Minnesota). All the sequences of the constructs were validated by DNA sequencing. Empty vectors without the cDNA insert were used as negative control 45. The plasmids were first transfected into 293FT cells for packaging and production of viral supernatants used to infect HL-1 cells. Transduced HL-1 cells were re-plated on 10 cm plates after 48 h of infection and selected using blasticidin (5 mg/ml) for 5-7 days to generate stable overexpression clones. The overexpression efficiencies were finally confirmed at both mRNA and protein levels 46.

### ELISA

Mouse Bax ELISA Kit (ab233624, Abcam), Mouse Bcl-2 ELISA Kit (NBP2-69946, Novus Biologicals), Mouse Glutathione Peroxidase 4 (GPX4) ELISA Kit (abx526114, Abbexa), Mouse PTX3 ELISA Kit (ab245713, Abcam), Mouse Thioredoxin Reductase 2 (TXNRD2) ELISA Kit (MBS9328396, MyBiosource), Mouse TNT/Troponin T ELISA Kit (F55154, LifeSpan BioSciences), Mouse Brain Natriuretic Peptide (BNP) ELISA Kit (CSB-E07971m, CUSABIO), and Mouse Creatine Kinase MB ELISA Kit (NBP2-74312, Novus Biologicals) were used to determine the activity/concentration of Bax, Bcl-2, Gpx4, Ptx3, Txnrd2, BNP, TnT, and CK-MB 47.

### Cell culture

HL-1 cells were cultured in DMEM (Gibco, Agawam, CA) supplemented with 10% fetal bovine serum (FBS, Invitrogen) at 37°C in a 5% CO_2_ atmosphere. To induce endotoxin-related cell damage, HL-1 cells were incubated with LPS (10 mg/ml) for 12 h 48.

### CCK-8 assay

Following experimental treatments, cell viability was conducted using a CCK8 Kit (44786, Dojindo, Wuhan, China) in HL-1 cells. Absorbance was measured at 450 nm 49.

### Statistical analysis

Data were statistically analyzed using SPSS software (IBM SPSS Statistics 25.0). Results were displayed as the mean ± SEM. Student's t-tests were performed to evaluate significant differences between two groups. More than two groups statistical analysis, we used One-way ANOVA and Bonferroni's post hoc test. P <0.05 was considered to indicate statistical significance.

## Results

### PHB2 overexpression attenuates LPS-induced myocardial depression

**Figure 1:**
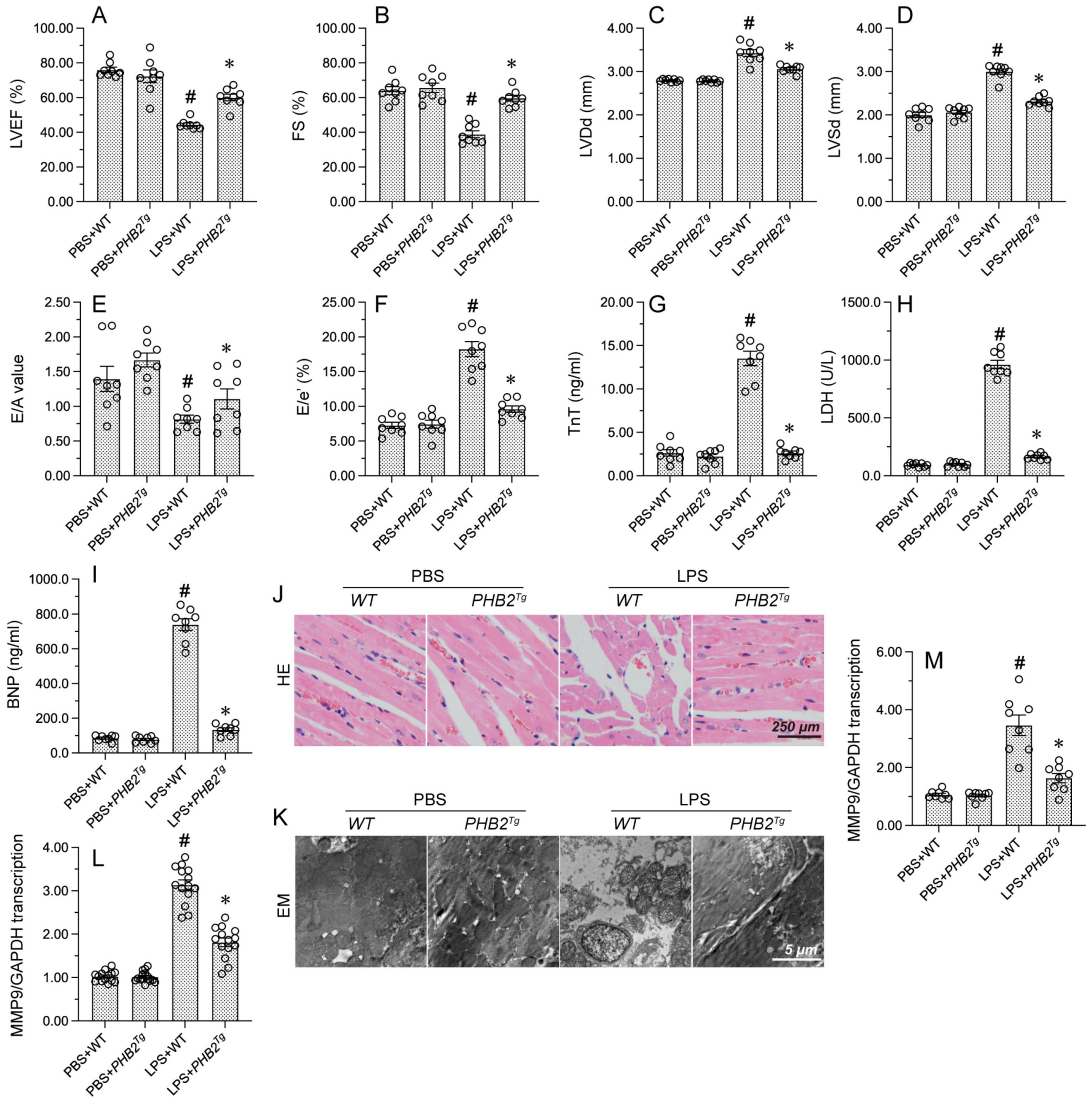
** PHB2 overexpression attenuates LPS-induced myocardial depression.** WT mice and PHB2 transgenic (PHB2^Tg^) mice were administered LPS to induce endotoxemia-related myocardial dysfunction.** (A-F)** Analysis of cardiac function via echocardiography. **(G-I)** Serum levels of TnT, CK-MB, and BNP were determined by ELISA. **(J)** Histopathological evaluation of cardiac tissue (HE staining).** (K)** Electron microscopy (EM) was applied to observe ultrastructural changes in myocardium. Cardiomyocyte swelling, fragmented cardiomyocyte nuclei, and twisted myocardial fibers are marked with red arrows. **(L, M)** RT-qPCR analysis of the transcription of CXCR7 and TNFα in cardiac tissue. #p<0.05 vs. PBS+WT group; *p<0.05 vs. LPS+WT group.

To evaluate whether PHB2 expression confers protective effects against endotoxemia-related myocardial dysfunction, LPS was injected into WT mice and PHB2 transgenic (PHB2^Tg^) mice and myocardial function was measured by echocardiography 24 h later. As expected, WT mice injected with LPS showed a decreased contractile function, evidenced by reductions in LVEF and fractional shortening (FS) (Figure 1A-1F). Suggesting dilatation of the ventricular chamber, higher LV dimensions (LVDs and LVDd) were observed in PHB2^Tg^ mice compared to WT control mice. LPS treatment also blunted cardiac relaxation capacity, as evidenced by impaired E/A and E/e', and these deleterious changes were also notably reduced in PHB2^Tg^ mice (Figure 1A-1F). To further assess LPS-induced myocardial damage, serum levels of cardiac injury markers, i.e. TnT, CK-MB, and BNP, were measured by ELISA in WT and PHB2^Tg^ mice. Consistent with cardiomyocyte dysfunction or death, elevated concentrations of TnT, CK-MB, and BNP were recorded in LPS-treated WT mice. Further indicating a cardioprotective effect, decreased levels of TnT, CK-MB, and BNP were instead observed in endotoxemic PHB2^Tg^ mice (Figure 1G-1I).

To confirm that the functional improvement and reduced damage of heart tissue evidenced in LPS-treated PHB2^Tg^ mice are associated with preserved myocardial structure, HE staining and electron microscopy (EM) were used to detect structural changes in the myocardium. Histological analysis via HE staining showed that after endotoxemia induction, myocardial swelling, nuclear fragmentation of cardiomyocytes, and myocardial fiber disarray were essentially absent in PHB2^Tg^ mice in comparison with WT mice (Figure 1J). Consistent with these observations, EM showed that mitochondrial swelling, cristae remodeling, and cardiomyocyte damage were largely attenuated in PHB2-overexpressing mice (Figure 1K). Since inflammation caused by the immune response to endotoxin is closely related to myocardial dysfunction, RT-qPCR was conducted to evaluate the effect of PHB2 overexpression on the transcription of cardiac inflammatory markers. Compared with PBS-treated mice, LPS administration significantly elevated the levels of CXCR7, a chemokine receptor that transduces signals from SDF-1α and I-TAC to recruit β-arrestins, and TNFα, a prominent pro-inflammatory cytokine, in the myocardium of WT mice. Notably, these changes were markedly suppressed in PHB2^Tg^ mice (Figure 1L and 1M). These results showed that PHB2 overexpression alleviates endotoxin-related myocardial depression through maintaining heart function and structure.

### PHB2 overexpression sustains cardiomyocyte viability and mitochondrial function in LPS-treated cardiomyocytes

**Figure 2:**
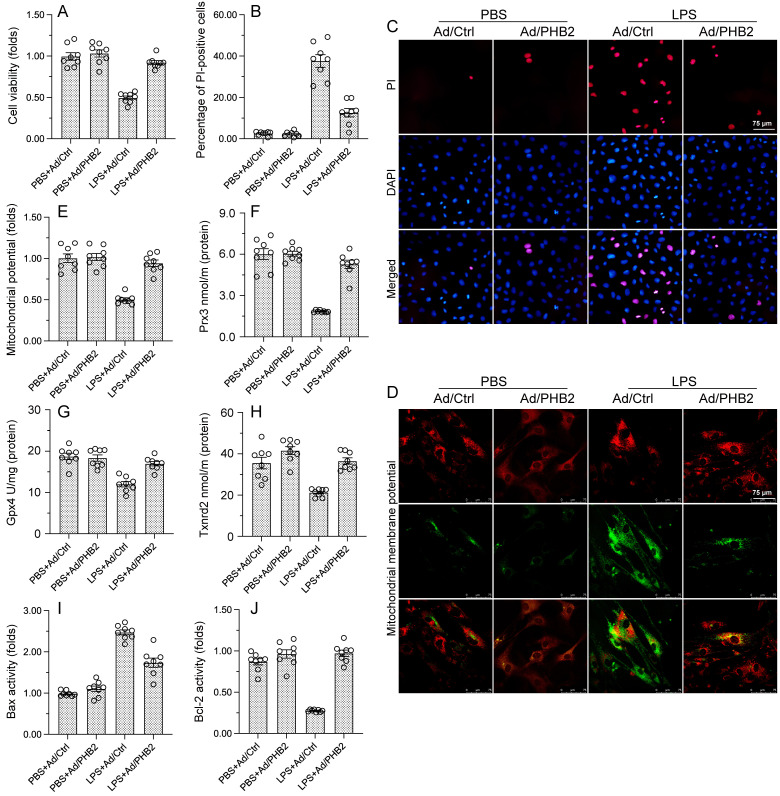
** PHB2 overexpression sustains cardiomyocyte viability and mitochondrial function during LPS exposure.** Cultured HL-1 cells transfected with PHB2 adenovirus (Ad/PHB2) or control adenovirus (Ad/Ctrl) were treated with LPS to simulate endotoxemia-mediated cardiac dysfunction *in vitro*. **(A)** Analysis of cell viability (CCK-8 assay). **(B, C)** Analysis of apoptosis by PI staining. **(D, E)** Evaluation of mitochondrial membrane potential in cells loaded with the JC-1 probe. **(F-H)** ELISA-based analysis of Gpx4, Prx3, and Txnrd2 protein concentrations in HL-1 cells. **(I, J)** ELISA-based analysis of Bcl-2 and Bax protein concentrations in HL-1 cells. #p<0.05 vs. PBS+WT group; *p<0.05 vs. LPS+WT group.

To assess the molecular bases of the beneficial effects of PHB2 overexpression on cardiomyocytes exposed to endotoxin, *in vitro* experiments were performed in HL-1 mouse cardiomyocytes transfected with PHB2-expressing adenovirus (Ad-PHB2) or control adenovirus (Ad-ctrl). Then, the effect of LPS on cardiomyocyte viability was determined by CCK-8 assay. Under control conditions, neither Ad-ctrl nor Ad-PHB2 influenced cardiomyocyte viability (Figure 2A). In contrast, following LPS exposure cell viability was significantly increased in Ad-PHB2-transfected cells (Figure 2A). In accordance with these findings, PI staining showed that the pro-apoptotic action of LPS was markedly neutralized by Ad-PHB2 transfection (Figure 2B and 2C).

Since cardiomyocyte viability is closely dependent on normal mitochondrial function, we wondered whether PHB2 overexpression-mediated cardiomyocyte survival in endotoxemia results from preserved mitochondrial homeostasis. Mitochondrial dysfunction characteristically involves mitochondrial membrane potential depolarization and reduced antioxidative capacity, leading to activation of the mitochondrial apoptotic pathway. JC-1 immunofluorescence assay showed that mitochondrial potential was reduced by LPS in Ad-ctrl-transfected cells, and this effect was negated in those overexpressing PHB2 (Figure 2D and 2E). In turn, ELISA results showed that the concentrations of mitochondrial antioxidant enzymes Gpx4, Prx3, and Txnrd2 were downregulated after exposure to LPS in control cells, and Ad-PHB2 transfection significantly reversed this expression trends (Figure 2F-2H).

The mitochondrial apoptotic pathway is triggered by upregulation of Bcl-2 and downregulation of Bax. ELISA showed that upon LPS exposure, Bcl2 activity was reduced, whereas Bax content was increased, in HL-1 cells transfected with Ad-ctrl. In contrast, Bcl2 activity and Bax expression remained at near-normal levels in Ad-PHB2-transfected cells (Figure 2I and 2J). These results suggested that PHB2 overexpression prevents endotoxin-induced cardiomyocyte death by preserving mitochondrial integrity.

### PHB2 phosphorylation promotes PHB2 mitochondrial localization and sustains mitophagy and UPR^mt^ in LPS-treated cardiomyocytes

**Figure 3:**
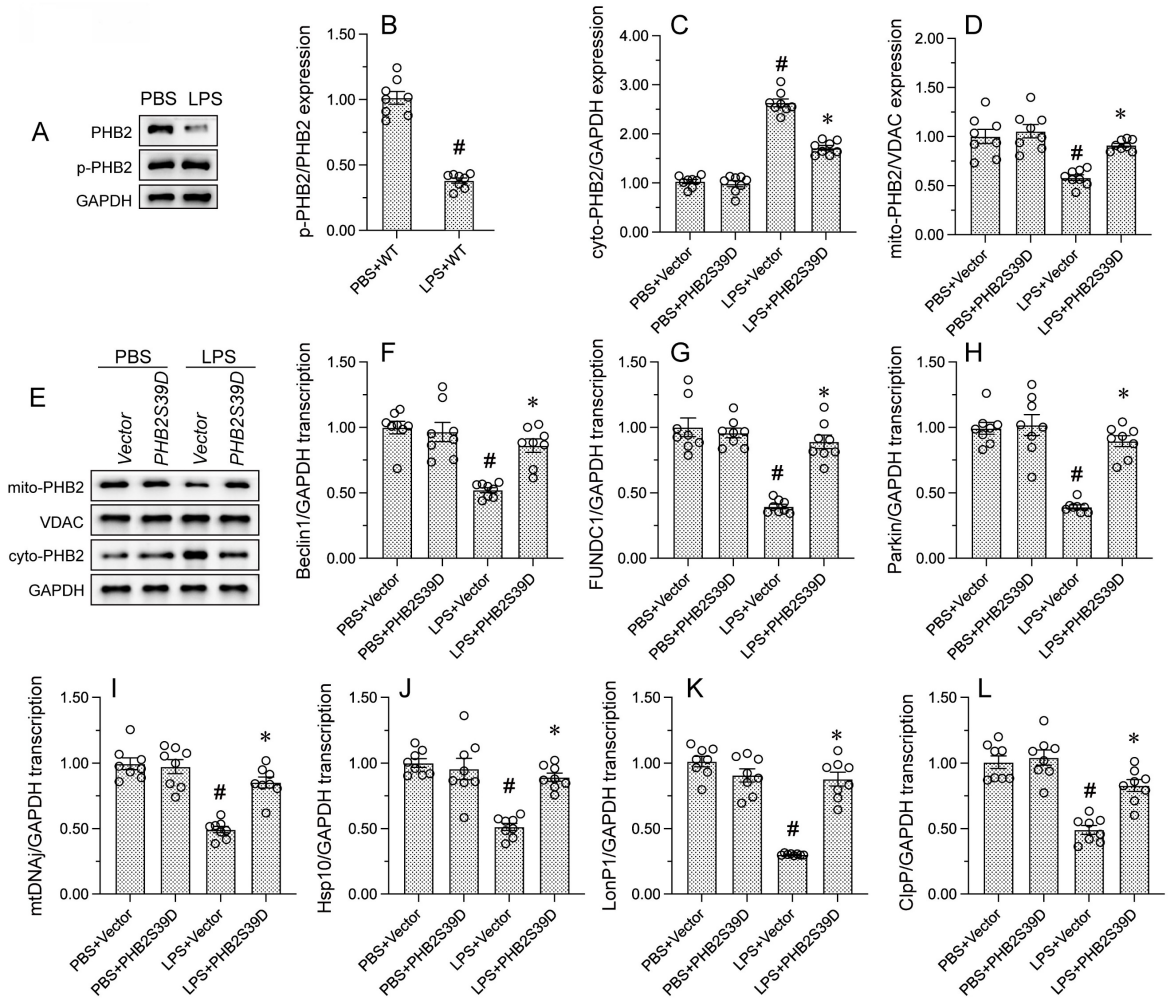
** PHB2 phosphorylation maintains mitochondrial localization of PHB2 and promotes mitophagy and UPR^mt^. (A, B)** Western blot analysis of PHB2-Ser^39^ phosphorylation in HL-1 cells. #p<0.05 vs. PBS+WT group.** (C-E)** Western blot analysis of mitochondrial and cytoplasmic PHB2 expression in HL-1 cells transfected with a phosphomimetic PHB2 mutant protein (PHB2S39D). #p<0.05 vs. PBS+Vector group; *p<0.05 vs. LPS+Vector group. **(F-H)** RT-qPCR analysis of transcriptional levels of the mitophagy-related markers Beclin1, FUNDC1, and Parkin in HL-1 cells. #p<0.05 vs. PBS+Vector group; *p<0.05 vs. LPS+Vector group. **(I-L)** RT-qPCR analysis of transcriptional levels of the UPR^mt^-related markers mtDnaJ, ClpP, LonP1, and Hsp10 in HL-1 cells. #p<0.05 vs. PBS+Vector group; *p<0.05 vs. LPS+Vector group.

Recent studies have found that PHB2 phosphorylation is required to maintain mitochondrial function by promoting mitochondrial localization of PHB2 50. Western blots showed that LPS induced PHB2 dephosphorylation at Ser^39^, and this effect was accompanied by reduced expression of mitochondrially-localized PHB2 (Figure 3A and 3B). To assess whether phosphorylation of PHB2 on Ser^39^ is required for PHB2 retention in mitochondria, HL-1 cells were transfected with a phosphomimetic PHB2-Ser^39^ (PHB2S39D) mutant protein. Western blot analysis showed that LPS-mediated cytoplasmic PHB2 accumulation was inhibited upon transfection of PHB2S39D (Figure 3C-3E). These results showed that LPS induces dephosphorylation of PHB2 at Ser^39^ and thus favors its release from the IMM.

To explore the outcome of mitochondrial PHB2 deficiency resulting from LPS-induced PHB2 dephosphorylation, we focused on mitophagy and UPR^mt^, two endogenous mitochondrial protective programs. RT-qPCR assays showed that LPS treatment reduced the mRNA abundance of mitophagy-related Beclin1, FUNDC1, and Parkin in HL-1 cells. Suggesting restored mitophagic activity, transfection of PHB2S39D was able to reverse the downregulation of these transcripts (Figure 3F-3H) Similarly, the transcription of genes related to UPR^mt^, namely mtDnaJ, ClpP, LonP1, and Hsp10, was downregulated by LPS in control HL-1 cells, and significantly normalized in those expressing PHB2S39D (Figure 3I-3L). These findings uncovered the functional importance of PHB2-Ser^39^ phosphorylation in sustaining mitophagy and UPR^mt^ in endotoxin-treated cardiomyocytes.

### LPS induces Pgam5-mediated PHB2 dephosphorylation

**Figure 4:**
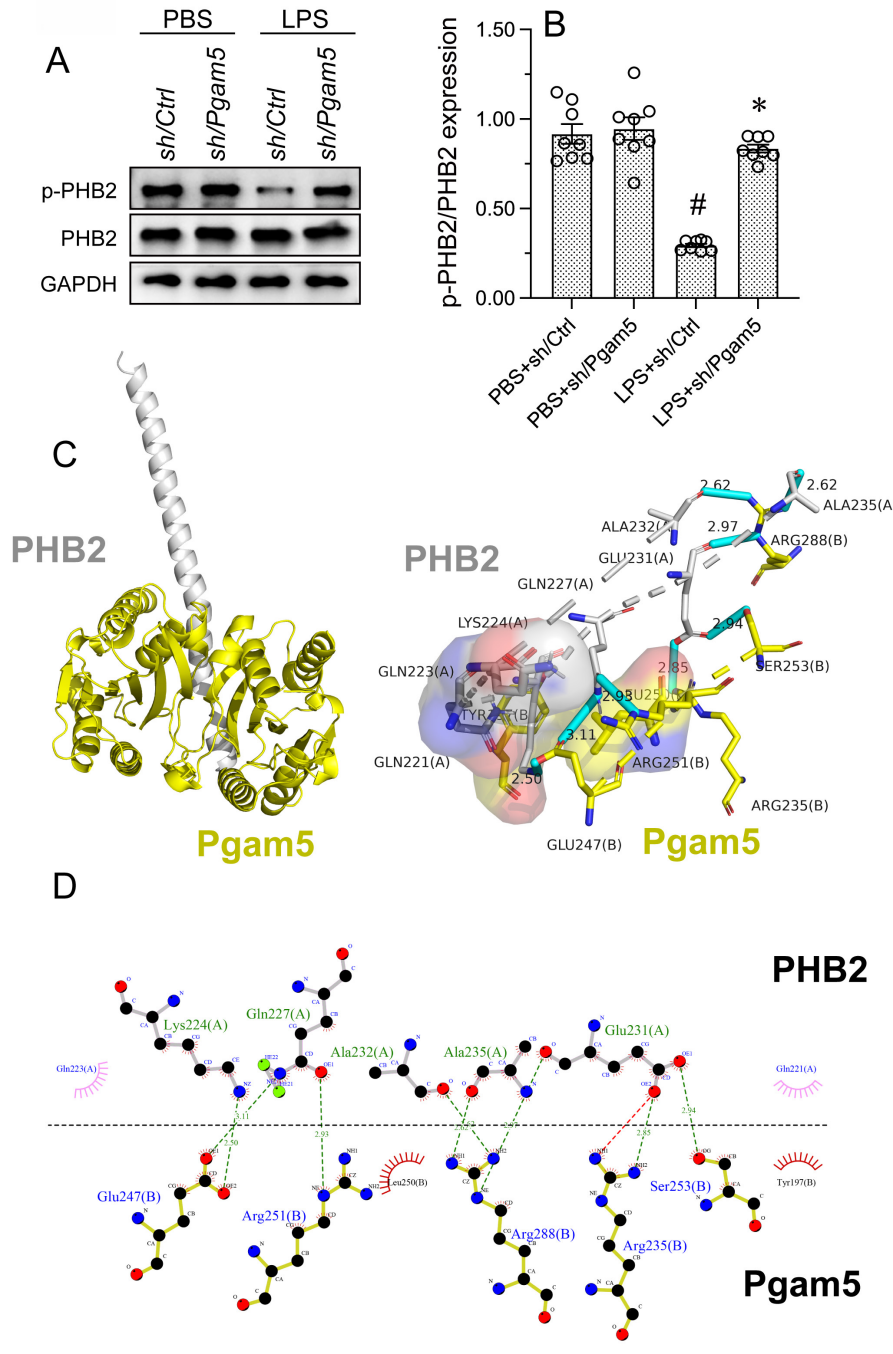
** LPS induces Pgam5-dependent PHB2 dephosphorylation.** Cultured HL-1 cells were transfected with shRNA against Pgam5 (sh/Pgam5) or control shRNA (sh/Ctrl) prior to exposure to LPS. **(A, B)** Western blot analysis of PHB2 phosphorylation in HL-1 cells. **(C, D)** Molecular docking analysis of the Pgam5-PHB2 interaction. The potential amino acid sites required for the binding between PHB2 and Pgam5 were analyzed. #p<0.05 vs PBS+sh/Ctrl group; *p<0.05 vs. LPS+sh/Ctrl group.

Previous studies have reported that Pgam5 induces PHB2 dephosphorylation in the setting of diabetic cardiomyopathy 35. Since Pgam5 ablation is able to protect the heart against inflammation-related injury, we asked whether Pgam5 contributes to endotoxin-related cardiomyocyte dysfunction by dephosphorylating PHB2. To assess this possibility, shRNA targeting Pgam5 (sh/Pgam5) was transfected into HL-1 cells and PHB2 phosphorylation was assessed by protein expression analysis. As illustrated in Figure 4A and 4B, LPS-induced PHB2-Ser^39^ dephosphorylation was nullified by sh/Pgam5.

We next conducted docking simulations to assess whether PHB2 dephosphorylation may result from direct interaction between Pgam5 and PHB2. This analysis predicted that Pgam5 may bind PHB2 through multiple amino acid sites (Figure 4C and 4D), with a minimum binding energy of -6.9 kcal·mol^-1^. The above data hence suggested that LPS induces PHB2 dephosphorylation through Pgam5.

### Pgam5 deletion restores mitophagy and UPR^mt^ in LPS-challenged cardiomyocytes

**Figure 5:**
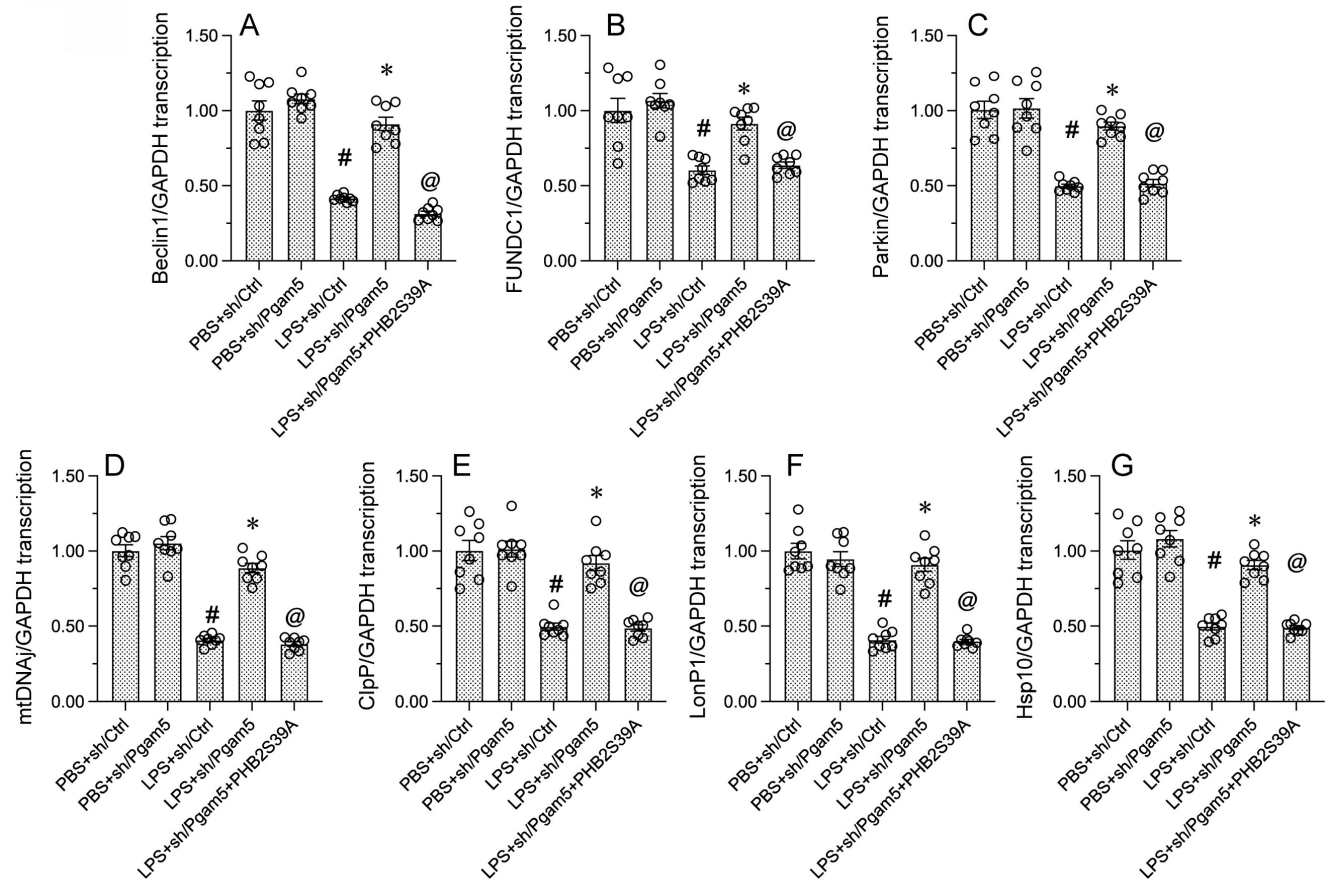
** Pgam5 deletion restores mitophagy and UPR^mt^ in LPS-challenged cardiomyocytes.** Cultured HL-1 cells were co-transfected with sh/Pgam5 (or sh/Ctrl) and phospho-defective PHB2S39A mutant protein prior to LPS exposure. **(A-C)** RT-qPCR analysis of Beclin1, FUNDC1, and Parkin transcription levels. **(D-G)** RT-qPCR analysis of the transcription of mtDnaJ, ClpP, LonP1, and Hsp10. #p<0.05 vs PBS+sh/Ctrl group; *p<0.05 vs. LPS+sh/Ctrl group; @p<0.05 vs. LPS+sh/Pgam5 group.

Given that Pgam5 knockdown was sufficient to prevent LPS-mediated PHB2 dephosphorylation, and that the latter correlates with delayed mitophagy and defective UPR^mt^, we investigated whether Pgam5 deficiency can normalize mitophagy and UPR^mt^ in LPS-treated cardiomyocytes. RT-qPCR assays showed that LPS-mediated transcriptional downregulation of the mitophagy markers Beclin1, FUNDC1, and Parkin was obviously reversed in HL-1 cells transfected with sh/Pgam5 (Figure 5A-5C). To verify that mitophagy restoration in Pgam5-deficient cells exposed to LPS results from inhibition of dephosphorylation of PHB2 on Ser^39^, we transfected a phospho-defective PHB2-Ser^39^ mutant protein (PHB2S39A) into sh/Pgam5-expressing HL-1 cells. Confirming the above assumption, PHB2S39A transfection abolished sh/Pgam5-mediated mitophagy activation, as evidenced by decreased transcription of Beclin1, FUNDC1, and Parkin, in LPS-treated cells (Figure 5A-5C). Similarly, the LPS-induced downregulation of gene transcripts related to UPR^mt^, namely mtDnaJ, ClpP, LonP1, and Hsp10, was abrogated in sh/Pgam5-transfected cells and reinstated upon co-expression of PHB2S293A (Figure 5D-5G). These results confirmed that Pgam5-mediated PHB2-Ser^39^ dephosphorylation is a critical determinant of LPS-mediated inactivation of both mitophagy and UPR^mt^ in cardiomyocytes.

### PHB2S293A transfection abolishes the protective effects of Pgam5 deletion on cardiomyocyte viability and mitochondrial function

**Figure 6:**
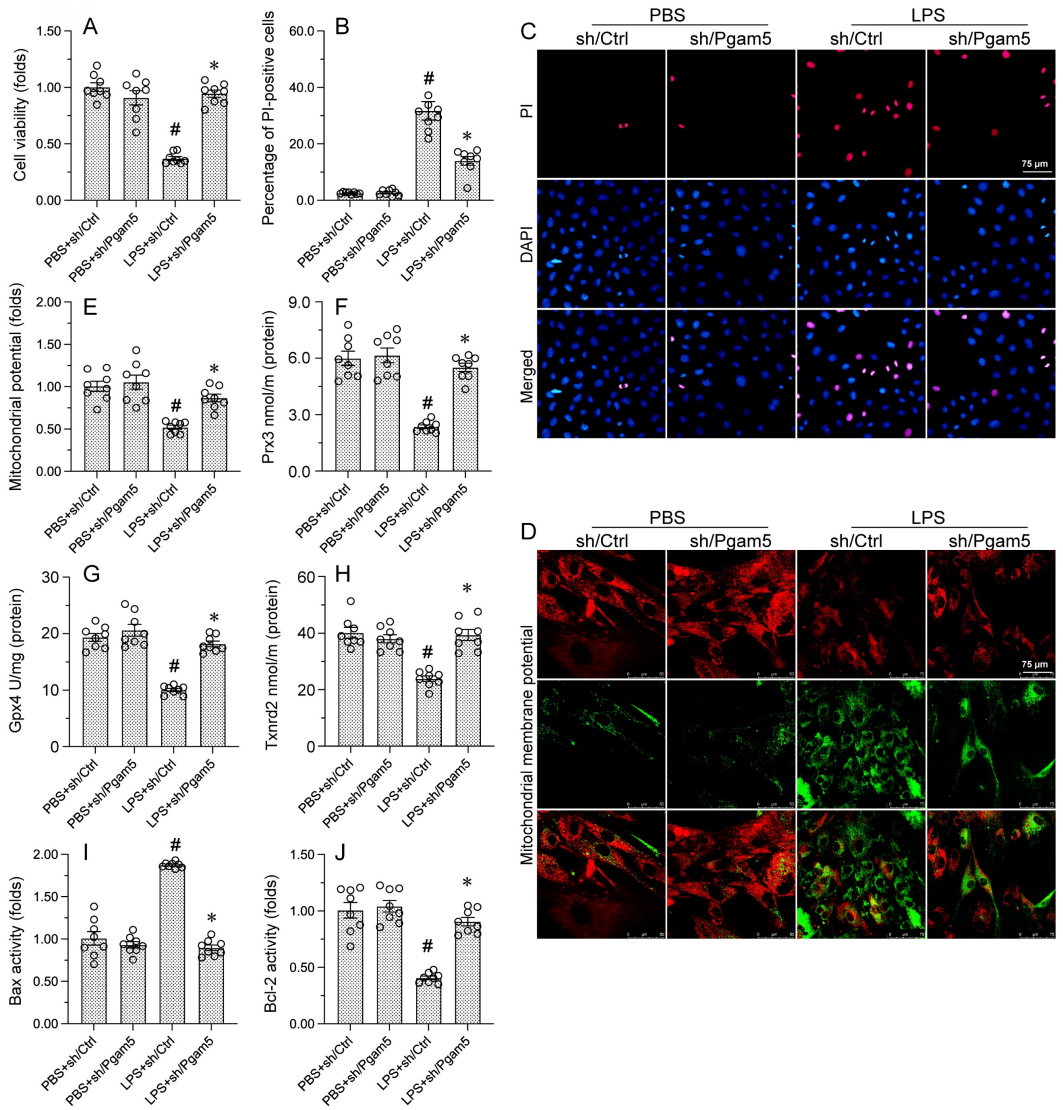
** PHB2S39A transfection abolishes the protective effects of Pgam5 deletion on cardiomyocyte viability and mitochondrial function. (A)** Cell viability analysis (CCK-8 assay) in HL-1 cardiomyocytes. **(B, C)** Analysis of apoptosis by PI staining. **(D, E)** Evaluation of mitochondrial membrane potential in HL-1 cells loaded with JC-1. **(F-H)** ELISA-based analysis of Gpx4, Prx3, and Txnrd2 protein concentrations. **(I, J)** ELISA-based analysis of Bcl-2 and Bax concentrations. #p<0.05 vs PBS+sh/Ctrl group; *p<0.05 vs. LPS+sh/Ctrl group; @p<0.05 vs. LPS+sh/Pgam5 group.

Since we showed that modulation of PHB2-Ser^39^ phosphorylation status by Pgam5 crucially influences two central c mechanisms, i.e. mitophagy and UPR^mt^^18^-^21^, ^51^, ^52^, in LPS-treated cardiomyocytes, we hypothesized that transfection with the phospho-defective PHB2S293A mutant construct would impair cardiomyocyte viability and mitochondrial homeostasis in LPS-treated cells expressing sh/Pgam5. Analysis of cell viability using CCK-8 assays showed that sh/Pgam5 transfection effectively rescued cardiomyocyte viability in LPS-treated HL-1 cells. Interestingly, the pro-survival effect of Pgam5 deletion was negated upon co-transfection with PHB2S39A (Figure 6A). Furthermore, PI staining showed that sh/Pgam5 transfection inhibited LPS-induced apoptosis, and this effect was nullified in cells expressing PHB2S39A (Figure 6B and 6C).

The impact of sh/Pgam5 and PHB2S39A expression on mitochondrial function was next assessed through analyses of mitochondrial membrane potential and antioxidant and apoptosis-related genes in LPS-challenged HL-1 cells. JC-1 staining assays showed that Pgam5 silencing prevented the reduction in mitochondrial membrane potential induced by LPS, and this protective effect was abolished upon transfection with PHB2S39A (Figure 6D and 6E). Similarly, following LPS exposure, expression levels of the mitochondrial antioxidant enzymes Gpx4, Prx3 and Txnrd2 attained near-normal values in cells transfected with sh/Pgam5, but remained instead downregulated in those transfected with PHBS39A (Figure 6F-6H). Consistent also with an anti-apoptotic effect, Pgam5 silencing normalized the balance between Bax and Bcl-2 expression, and this effect was negated upon expression of PHB2S39A (Figure 6I and 6J). These results verified our hypothesis that Pgam5-mediated dephosphorylation of PHB2-Ser^39^ mediates mitochondrial dysfunction and apoptosis in endotoxin-exposed cardiomyocytes.

### Pgam5 deletion improves myocardial function in endotoxemic mice

**Figure 7:**
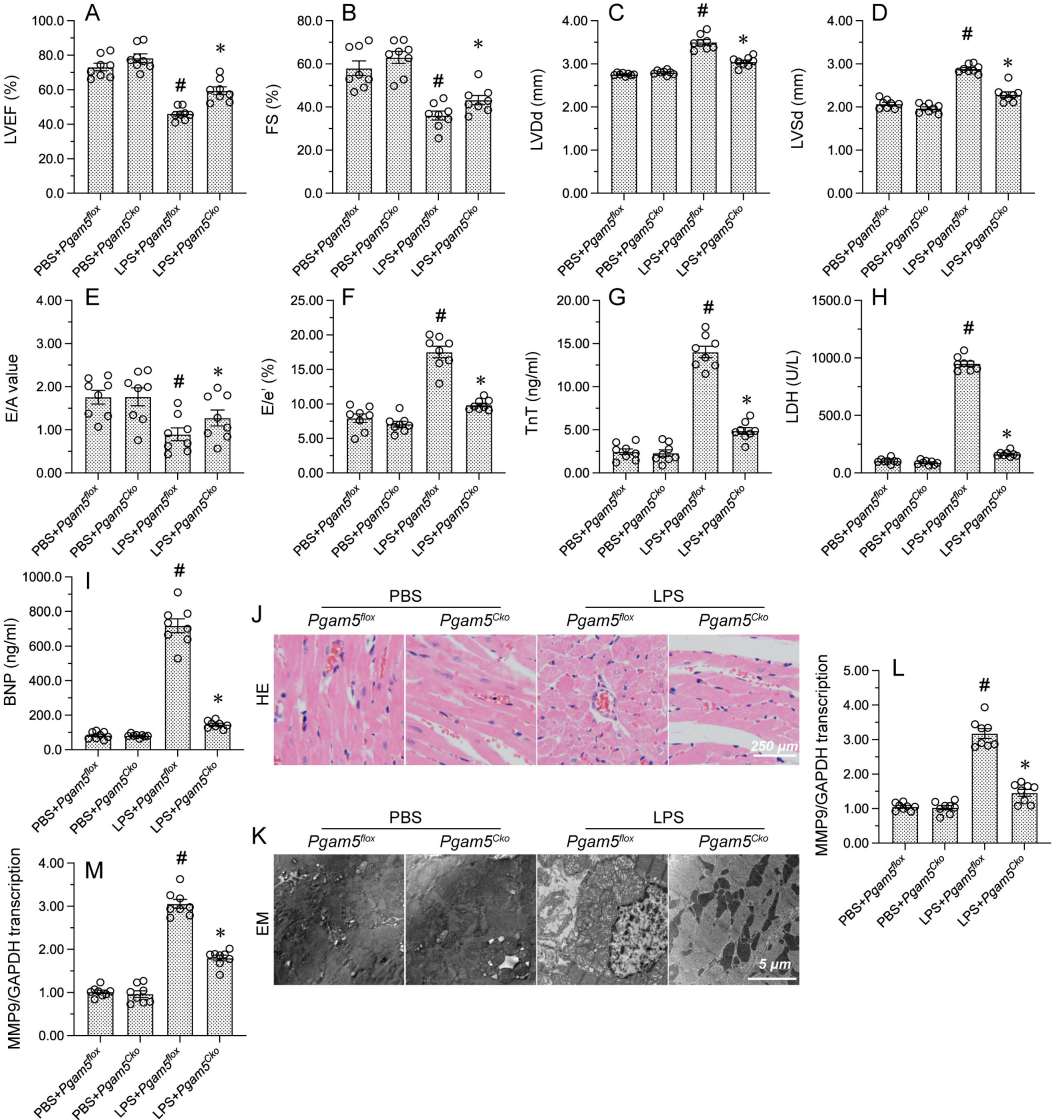
** Pgam5 deletion improves myocardial function in LPS-treated mice.** WT mice and cardiomyocyte-specific Pgam5 knockout (Pgam5^Cko^) mice were administered LPS to induce endotoxemia-related myocardial dysfunction.** (A-F)** Analysis of cardiac function via echocardiography. **(G-I)** Serum levels of TnT, CK-MB, and BNP were determined by ELISA. **(J)** Cardiac histopathology analysis by HE staining.** (K)** Electron microscopy (EM) was applied to analyze ultrastructural changes in myocardium. Cardiomyocyte swelling, fragmented cardiomyocyte nuclei, and twisted myocardial fibers are marked with red arrows. **(L, M)** RT-qPCR analysis of CXCR7 and TNFα mRNA levels in heart tissues. #p<0.05 vs. PBS+*Pgam5^Cko^* group; *p<0.05 vs. LPS+*Pgam5^Cko^* group.

To investigate the mechanisms of Pgam5 in endotoxemia-related myocardial depression, cardiomyocyte-specific Pgam5 knockout (*Pgam5^Cko^*) mice were exposed to LPS. Echocardiography showed that myocardial contractile function was improved in *Pgam5^Cko^* mice compared with WT mice, as evidenced by increased LVEF, FS, and LVDs. Similarly, the detrimental effects of LPS on E/A, E/e', and LVDd were largely abrogated in *Pgam5^Cko^* mice (Figure 7A-7F). Further evidence that Pgam5 deficiency attenuates LPS-related myocardial injury was obtained by ELISA, which demonstrated markedly reduced serum levels of TnT, CK-MB, and BNP in *Pgam5^Cko^* mice compared to WT mice (Figure 7G-7I).

In addition to the functional improvement described above, improved cardiac structure, evidenced by preserved nuclear morphology and myocardial fiber organization, were observed in endotoxemic *Pgam5^Cko^* mice (Figure 7J). In line with these findings, EM analysis showed no evidence of myocardial fiber swelling and mitochondrial rupture in these mice (Figure 7K). Furthermore, suggesting reduced inflammation, after LPS exposure the transcription of CXCR7 and TNFα in heart tissue was maintained at near-normal levels in *Pgam5^Cko^* relative to WT mice (Figure 7L and 7M). Our findings proposed that *Pgam5* ablation greatly attenuates endotoxin-mediated cardiac dysfunction in mice.

## Discussion

Herein, we report on the complex relationship between the mitochondrial serine/threonine-protein phosphatase Pgam5 and PHB2, a component of the mitochondrial prohibitin complex, in endotoxemia-mediated myocardial dysfunction. Our findings suggest that Pgam5 deletion stabilizes myocardial function during LPS stress through maintaining mitochondrial function in cardiomyocytes. Based on *in vitro* and *in silico* analyses, we concluded that under endotoxemia conditions, Pgam5 directly interacts with PHB2 and induces PHB2 dephosphorylation at Ser^39^. Notably, the latter prevents PHB2 mitochondrial import and leads to mitochondrial dysfunction and cardiomyocyte death by inhibiting mitophagy and the UPR^mt^. Consistent with the above findings, global overexpression of PHB2 in mice or transfection of a mutant PHB2 protein containing a phosphomimetic substitution of Ser^39^ in cultured cardiomyocytes effectively reduced LPS-mediated myocardial injury and cardiomyocyte death.

The three main findings of the present study can be summarized as follows: 1) Pgam5 activation is an initial upstream signal in endotoxemia-related myocardial depression through a mechanism involving PHB2 dephosphorylation; 2) LPS-induced, Pgam5-dependent PHB2 dephosphorylation impedes PHB2 retention in mitochondria, which inactivates stress-related mitophagy and UPR^mt^; 3) Aberrant mitophagy and UPR^mt^ are associated with mitochondrial dysfunction and cardiomyocyte death, which correlate with disrupted myocardial structure and function characteristic of septic cardiomyopathy. These findings indicate that Pgam5 and PHB2 are potential interventive targets for the treatment of endotoxemia-related myocardial depression. Based on the functional impact of the Pgam5-PHB2 interaction on cardiomyocyte homeostasis, we further suggest that therapeutic strategies aiming at stimulating mitophagy and UPR^mt^ may be useful to alleviate endotoxemia-induced cardiac injury.

Previous studies have described in detail the pathological contribution of Pgam5 to inflammation-related diseases. For example, Pgam5-related oxidative stress and inflammation leading to programmed necrosis of liver cells was demonstrated in a mouse model of acute liver injury 53. Experiments in mice showed also that during the development of acute endotoxemic lung injury, NR4A1-mediated activation of Pgam5 contributes to decreased mitochondrial fusion and necroptosis of lung cells. In a mouse model of septic kidney injury, loss of Pgam5 inhibited inflammation by preventing Bax dephosphorylation-mediated release of mitochondrial DNA 54-56. Similarly, inhibition of Pgam5 was shown to alleviate experimental autoimmune encephalomyelitis by suppressing necroptosis of activated microglia 57. In mice subjected to myocardial ischemia/reperfusion injury, suppression of Pgam5 interrupted Keap1-mediated Bcl-xL degradation and promoted cardiomyocyte survival 58. In accordance with the above findings, our study suggests critical pro-inflammatory and pro-apoptotic roles of Pgam5 in the development endotoxemia-mediated myocardial depression. Given the central contribution of Pgam5 to numerous inflammation-related diseases, the design of novel drugs targeting Pgam5 seems to be a critical method for the management of endotoxemia-induced organ dysfunction.

PHB2 contributes to maintaining mitochondrial integrity, especially by supporting the functionality of the IMM 38. Since mitochondrial oxidative stress primarily involves deficiencies in the oxidative phosphorylation cascade, which takes place in the IMM, the stability of PHB2 is closely associated with mitochondrial energetics and metabolism. Thus, loss of PHB2 was shown to contribute to heart failure by suppressing fatty acid oxidation in cardiomyocytes 31. Importantly, PHB2 is associated with PINK1/Parkin mitophagy 59, and is required for the interaction between mitochondria and the autophagosome marker LC3 24. In this regard, PHB2 knockdown was reported to attenuate angiotensin II-induced cardiac fibrosis by inhibiting mitophagy in cardiac fibroblasts 30. Recent data highlighted an indispensable impact of PHB2 in attenuating mitochondrial fission and maintaining mitochondrial membrane potential 60. Notably, stimulation of PHB2-dependent mitophagy has been associated with delayed heart aging in mice 61. In addition to its multifaceted role in mitochondria biology, several studies have uncovered the relationship between PHB2 and inflammation. PHB2 was shown to govern fatty acid composition in macrophages 62. In line with the present findings, which showed that overexpressing PHB2 is an effective way to reduce myocardial inflammation and normalize heart performance under endotoxemia conditions, evidence from rodent models of arthritis 63, spondylarthropathies 64, ulcerative colitis 65, and endotoxin-mediated acute lung injury 66 highlighted also the functional importance of PHB2 in neutralizing abnormal inflammatory responses.

Importantly, our results showed that Pgam5-mediated dephosphorylation of PHB2-Ser^39^ is a novel pathological alteration that contributes to endotoxemia-mediated mitochondrial damage and cardiomyocyte death by preventing PHB2 mitochondrial recruitment. In this regard, current evidence indicates that mitochondrial PHB2 expression seems to be a prerequisite for the activation of both mitophagy and the UPR^mt^, two essential mitoprotective mechanisms 67-69. Therefore, our results broaden previous findings that point to PHB2 phosphorylation as a promising target to ensure proper function of mitochondrial protective programs upon cellular stress.

Mitophagy promotes removal of damaged mitochondria through lysosomal degradation, whereas UPR^mt^ promotes protease-mediated degradation of abnormal mitochondrial proteins 70-72. Extensive evidence supports the cardioprotective actions of mitophagy and UPR^mt^ in heart disease. Increased mitophagy is able to attenuate myocardial ischemia/reperfusion injury through sustaining mitochondrial metabolism 73, 74. Similarly, the progression of diabetic cardiomyopathy can be delayed by mitophagy activation, linked to decreased mitochondrial oxidative stress and improved cardiomyocyte metabolism 43, 46, 75-77. Research has shown that LPS-induced myocardial depression is associated with reduced mitophagy, and pharmacological activation of mitophagy is able to improve the function of the septic heart 51, 78. Similarly, cardiac ischemia/reperfusion injury is also alleviated by activation of the UPR^mt^, in connection with improved mitochondrial function 48, 79-81. Our present data revealed that both mitophagy and the UPR^mt^ are under the control of the Pgam5/PHB2 axis, suggesting that a unique signaling pathway may function upstream of these two processes. As recent studies have highlighted potential interactive effects between mitophagy and UPR^mt^ 48, additional experiments may help further decipher the complex mechanisms underlying dysfunctional mitophagy and UPR^mt^ in septic cardiomyopathy.

In conclusion, our findings revealed that Pgam5-mediated dephosphorylation of PHB2 critically contributes to endotoxemia-related cardiac dysfunction by preventing PHB2 mitochondrial import, which is required for activation of both mitophagy and UPR^mt^. Accordingly, either cardiomyocyte-specific Pgam5 deficiency or global PHB2 overexpression were able to improve mitochondrial function and reduce cardiomyocyte death in endotoxin-treated mice. Our findings thus identified the Pgam5/PHB2 interaction as a promising target for the treatment of endotoxemia-related cardiac dysfunction.

## Supplementary Material

Supplementary table.
