# Supplementary material for: Pgam5-mediated PHB2 dephosphorylation contributes to endotoxemia-induced myocardial dysfunction by inhibiting mitophagy and the mitochondrial unfolded protein response
Source: Int J Biol Sci. 2023 Aug 28;19(14):4657–71. doi: 10.7150/ijbs.85767 (PMC10535708; doi:10.7150/ijbs.85767)
Supplement: Supplementary file 1 — Supplementary table. [file ijbsv19p4657s1.pdf]

<u>Primer sequences for RT-PCR</u>

**Gene**	**Forward (5’-3’)**	**Reverse (5’-3’)**
**mtDNAj (mouse)**	AGTCACCCACACAAGCACTG	CCAGCCTCTCGCCTATCC
**ClpP (mouse)**	CACAGACATCGCCATCCA	TCCCTCTCCATTGCTGACTC
**LonP1 (mouse)**	GGTTGAGAATGTAGCCCATGA	CGATGATATCCCGAATGGTC
**Hsp10 (mouse)**	GGCCCGAGTTCAGAGTCC	TGTCAAAGAGCGGAAGAAACTT
**Fundc1 (mouse)**	AGACACCACTGGTGGAATCGAG	CCTTCTGGAATAAAAATCCTGCAC
**Parkin (mouse)**	TGTCCCAACTCCCTGATTAAAG	ACAGCACACCTCCCATTTG
**Beclin-1 (mouse)**	CTGCACAGGGAACACAGCAA	GCCAGCGGCTATGAGAGAA
